# Retrospective Analysis of Metabolic Syndrome: Prevalence and Distribution in Executive Population in Urban Pakistan

**DOI:** 10.1155/2012/649383

**Published:** 2012-09-05

**Authors:** Niloufer Sultan Ali, Ali Khan Khuwaja, Adnan-ur-Rahman, Kashmira Nanji

**Affiliations:** ^1^Department of Family Medicine, Aga Khan University Hospital, Aga Khan University, Stadium Road, P.O. Box 3500, Karachi 74800, Pakistan; ^2^Jinnah Postgraduate Medical College, Jinnah Postgraduate Medical Centre (JPMC), Rafiquee Shaheed Road, P.O. Box 3937, Karachi 74800, Pakistan

## Abstract

*Background*. Metabolic Syndrome (MetS) is a major public health concern. *Objective*. The aim of this study was to estimate the frequency of MetS, its components, and factors associated with MetS amongst apparently healthy individuals in Pakistan. *Methods*. A retrospective cross-sectional study was conducted at the executive Clinics of Aga Khan Hospital, Pakistan. Medical records of patients aged ≥18 years visiting the clinics from July 2011 to December 2011 were consecutively reviewed. Records in which either MetS components data or 10% of overall data was missing were excluded. A total of 1329 participants' records was included in final analysis. Data was analyzed using SPSS version 19 and multivariable logistic regression was used to identify the factors associated with MetS. *Results*. A total of 847 (63.7%) participants had MetS; mean age of the participants were 47.6 ± 11.6 years. About 70.4% were males and 29.6% were females. Approximately 70% of participants had BMI ≥25 kg/m^2^. MetS was associated with male gender (AOR = 2.1; 95% C.I: 1.6–3.2) and history of diabetes among parents (AOR = 3.0; 95% C.I: 1.6–6.0). *Conclusion*. This study shows that a large proportion of population has MetS and is overweight or obese. This requires urgent interventions on part of health care providers' especially family physicians. Educating masses about life style factors can make a difference. Further researches on this issue are warranted.

## 1. Introduction

Globally, Metabolic Syndrome (MetS) has become a public health concern and is a major cause of morbidity and mortality. Many studies have provided strong evidence for the association of MetS with acute coronary syndrome [1–4]. In health care services the value of MetS derives largely from its potential to reduce the risk of cardiovascular disease in the general population by treating the disease [5].

There has been much a debate on the definition of MetS. However, recent definition of MetS given by American heart association and the National heart, Lung and Blood Institute (AHA/NHLBI) in 2005 declared that a clinical diagnosis of MetS can be established if any three of the following factors are present, elevated triglyceride level (TG): elevated waist circumference, decreased HDL-cholesterol (HDL) level, elevated fasting glucose, and elevated blood pressure [6]. 

The estimated prevalence of MetS in general population is between 17 and 25% [7]. Various studies have demonstrated that the major causes leading to MetS are insulin resistance, obesity, and genetic predisposition [8]. Several cross-sectional studies have established that overweight and obesity are clearly associated with risk factor components that make up MetS [9]. 

A recent study from Sweden has shown that presence of MetS can give essential prognostic information of cardiovascular mortality if the current status of risk factors is known in an individual [2]. Recently questions have been raised about the role of MetS as a separate entity, associated with a greater risk of development of cardiovascular disease. Indo-Asian population has highest risk of developing coronary artery disease in the world, thus it is not surprising that it has become a common cause of death in this subcontinent [10, 11]. With increasing urbanization the risk of developing MetS and its associated risk factors like ischemic heart disease is only likely to show an upward trend.

The incidence of Type 2 Diabetes Mellitus (T2DM) has been increasing in the last decades and is projected to rise from 171 million in 2000 to 366 million in 2030 [12]. In Pakistan, about 10% of adult population is having diabetes, ranking sixth with an increasing trend [13]. It is projected that if the situation persists, by the year 2030 Pakistan will rank the 4th in the world for the burden of diabetes [13]. Several population studies have indicated that metabolic syndrome is a risk factor for the development of T2DM [8, 14, 15].

Studies have shown that unlike European countries the risk of developing cardiovascular disease and its associated risk factors like hypertension, dyslipidemia is nearly equivalent among men and women in Asian countries [16]. A very often occurring point in the prevalence of MetS is that it shows age dependence whereas its prevalence has been shown to increase with increasing age [17]. Among South Asians cardiovascular risk factors are seen at a younger age and the reason for this is still unclear [18]. 

Recently some controversies were raised by Kahn regarding the definition of MetS which implies that the factors included in the definition of MetS should have greater predictive power than all other combinations [19]. Nonetheless, there is no obvious evidence which shows this. 

Family physicians/general practitioners mainly focuse on holistic and integrated care with health promotion and disease prevention: the core of their practice. Usually they are the first contact with the patients, hence, awareness regarding MetS and the individual risk of each component is vital so that individualized treatment/prevention strategies can be implemented. Therefore, this study was designed to estimate the frequency of MetS, its components, factors associated, and clustering of different components of MetS amongst apparently healthy individuals in Pakistan.

## 2. Material and Methods

A retrospective cross sectional study was done at the executive Clinics of the Aga Khan University Hospital Karachi, Pakistan (AKUH). The Aga Khan University is a not-for-profit, tertiary care hospital. Medical records of all patients aged 18 years onwards who came for their routine annual physical checkups over a period of six months at executive clinics of AKUH from July 2011 to December 2011 were consecutively reviewed. Patients for which either MetS components (BMI, FBS, HDL, BP, TRIG) data or 10% of overall data was missing were excluded from the study. A total of 1500 medical records were reviewed. 171 patients' data were incomplete and therefore were excluded and remaining 1329 patients' records were included in final analysis.

 The routine examination at the executive clinics, AKUH, includes a detailed physical examination comprising height, weight, blood pressure, thorough general and physical examination along with complete blood picture, erythrocyte sedimentation rate, fasting blood sugar, lipid profile, liver function tests, creatinine, uric acid, chest X-ray, and ECG.

A structured questionnaire was formulated to extract the information. These questionnaires were filled by a medical graduate specifically trained for the task and it was composed of two sections. Section A included questions on demographic detail of the participants (age, gender). Section B dealt with the risk factors of MetS including BMI, blood pressure, fasting blood sugar, triglycerides, and HDL. This section also included questions on family history of CVD among parents, sibling, and children. The study was reviewed and approved by the Family Medicine's Departmental Research Committee of The Aga Khan University Hospital, Karachi, Pakistan. 

According to the new International Diabetes Federation definition, for a person to be defined as having the metabolic syndrome they must have the following [20].

**Central Obesity**

Males ≥90 cm and females ≥80 cm (based on Asian Indian, Malay & Chinese population). The new International Diabetes Federation definition suggests that If BMI is >30 kg/m², central obesity can be assumed and waist circumference does not need to be measured. In this study we used Asian cutoff for obesity that is, ≥25 kg/m^2^.

Also they must have any two of the following four factors:

1. raised triglycerides: ≥150 mg/dL (1.69 mmol/L);
2. reduced HDL cholesterol: <40 mg/dL (1.04 mmol/L) in men and <50 mg/dL (1.29 mmol/L) in women;
3. raised blood pressure: ≥130/85 mm Hg;
4. raised plasma fasting glucose: ≥100 mg/dL (≥6.1 mmol/L).

Statistical analysis was carried out using SPSS software (Statistical Package for the Social Sciences, version 17). Proportions were reported for all the variables such as age, gender, and components of MetS. Univariate and multivariate logistic regression was done to identify the factors associated with MetS. The results are reported in form of odds ratio and 95% confidence intervals. A *P* value of <0.05 was considered statistically significant throughout the study.

## 3. Results

A total of 1329 patients' records were included in the final analysis and for missing data we averaged estimates of the variables to give a single mean estimate. The medical record indicates that out of the total patients 936 (70.5%) were males and 393 (29.5) were females. The mean age of the patients was 47.6 ± 11.6 years. 

Table 1(a) depicts the age- and gender-specific percentages of individual component of MetS. A total of 847 participants had MetS, thus showing a prevalence of 63.7%. Almost 70% of the patients had BMI over 25. As compared to females, males had higher prevalence of BMI over 25 (70% v/s 30%). Over two-thirds of the patients had raised plasma blood glucose. However, one-third (33%) of the total female population had reduced HDL levels. 

Table 1(b) shows the age- (less than 40 years) and gender-specific component of MetS. In all, 23.3% of the populations were aged less than 40 years, with 68.1% males and the rest were females. About 34.8% of the patients had raised triglyceride levels, while 65.6% of the males and 34.4% of the females less than 40 years had reduced HDL levels (*P* = 0.04). The *P*-values were significant for all components, suggesting there was significant difference among males and females with respect to the components of MetS. 

Table 1(c), presents the age and sex specific distribution of components of MetS, who are more than 40 years old. About three fourth of the total patient population were more than 40 years of age. Majority of the patients (71.7%) had BMI >25 and all the results were statistically significant at 5% significance level (*P* < 0.05) except raised blood pressure (*P* = 0.59).

The association of demographic and family factors with MetS is presented in Table 2. The odds of having MetS were 2 times higher among males (AOR: 2.1; 95% C.I: 1.6–3.2) as compared to females. Those patients who had past history of diabetes were 5 times at risk of developing MetS as compared to others when adjusted for other variables in the model (AOR: 5.3; 95% CI: 1.0–74) and was highly significant (*P* < 0.001). About 60% of the patients who had MetS had family history of diabetes among parents, while about two-third MetS patients had family history of diabetes among siblings (AOR: 3.0; 95% C.I: 1.6–6.0). The other variables which were statistically insignificant at the multivariate regression were family history of CVD among parents and siblings. About 41% of the patients who had MetS; had 5 or less years of education and the maximum risk was also observed amongst them (AOR: 3.0; 95% CI: 2.4–3.8). 

**Figure 1:**
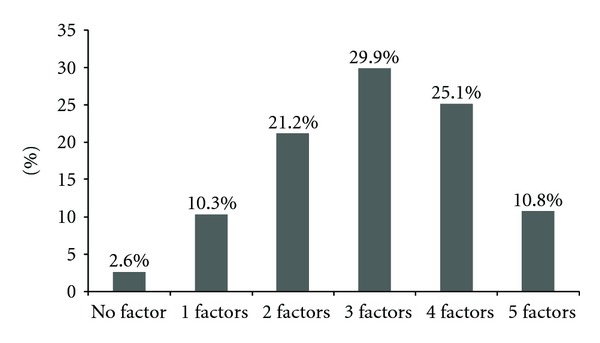
Pattern of appearance of MetS components among study participants.

The pattern of appearance of MetS components among study participants is depicted in Figure 1. Only 2.6% of the participants had no risk factor of MetS, while around 11% of the participants had 5 or more risk factors. Almost 30% of the participants had 3 risk factors, whereas, 10% and 21% of the participants had 1 and 2 risk factors of MetS respectively.

## 4. Discussion

In this study the prevalence of MetS was found to be 63.7% among the study participants. The results also highlight that BMI over 25 was the most common (70.3%) risk factor present among the cases of MetS. This prevalence of MetS is almost twice as high as in the United States as determined in NHANES data [21]. The major reason for this variation can be the fact that in this study we used BMI ≥25 kg/m^2^ for diagnosis of MetS and due to this a large proportion of participants were included in the definition of MetS. According to IDF, a BMI cutoff of ≥30 kg/m^2^ can be used for diagnosing MetS [20], nonetheless in Asian population the cutoff for obesity is ≥25 kg/m^2^, which was used in this study [22]. However, Asian cutoff of BMI for diagnosis of diabetes and CVD is ≥23 kg/m^2^ due to the difference in adiposity, as Asians tend to have more central obesity than other populations [22]. Though analysis was also performed at BMI cutoff of ≥23 kg/m^2^ to observe any change in the prevalence of MetS. Nonetheless there was no difference in prevalence of MetS when the cutoff ≥23 kg/m^2^ was used. 

The prevalence of MetS in Pakistan is showing an upward trend [23]. The results of this study cannot be compared to other studies conducted in Pakistan. As these studies have shown contrasting prevalence of MetS ranging from as low as 20% and as high as 85% [23–25], there were differences in definitions of MetS and population selection, that is, participants with comorbidities such as T2DM. A study by Mohsin et al. found a high prevalence of 85% and was conducted on type 2 diabetics [25]. Results from other populations reported the prevalence to be between 70 to 80% among Caucasians with T2DM [26] and 75.6% among Chinese diabetics [27]. This high prevalence 63.4% of MetS that we observe in the study can be attributed to 43% of the patients who had past history of T2DM.

It is well reported that the risk factors which are most frequently present in MetS cases include obesity, coronary heart disease, sedentary lifestyle, aging, and lipodystrophy [28, 29]. 

Various studies reveal that the components which make up MetS vary in the rates in which they occur in different populations [18, 19]. The Seychelles study conducted on 1255 participants reported that adiposity and high blood pressure are most common occurring components of MetS [30]. However, in the present study BMI in 70% and high fasting blood glucose in 62% of the study participants were the most prevalent components. This is not surprising as it has been evident that central obesity precedes the appearance of other MetS components and it also plays a crucial role in the development of MetS [31]. Moreover, in Asian population central obesity is found to be more common [22]. An analysis of the National Health Survey of Pakistan revealed that 25% of our population is obese [22]. Latest researches have now shown that although obesity is an intriguing factor of MetS there are some phenotypes which alters this definition [32]. Despite having BMI exceeding 30 kg/m^2^, individuals are relatively insulin sensitive and lack most of the metabolic abnormalities typical of obese individuals [32]. These individuals' phenotypes separate obesity from its usual metabolic consequences and demands more focus on clustering of the risk factors. 

The incidence of T2DM worldwide is rising exponentially due to increase in sedentary life style leading to central abdominal obesity [22]. T2DM is one of the most common chronic diseases worldwide and the fourth or fifth leading cause of death in the developed world [12, 13]. There is ample data to suggest that presence of MetS intensifies the risk of developing T2DM to manyfold [14, 15]. “Clustering” of metabolic abnormalities (hypertension, hyperinsulinemia hypertriglycemia, hyperglycemia) that occur in the individual appears to confer a substantial additional risk for developing future T2DM, which would add to the 230 million people worldwide who already have diabetes [12, 18]. 

A prospective cohort study conducted on 2,924 patients found that the presence of MetS in patients was associated with a four- to sixfold increased risk of future T2DM [33]. Another study suggested that people with metabolic syndrome have a fivefold greater risk of developing T2DM [34]. In this study we found that about 62.8% of the total population had impaired fasting glucose, which is an important indicator to predict T2DM. These results are also consistent with a study conducted in Africa which concluded that a large proportion of patients with impaired glucose tolerance had MetS [30]. 

Studies have presented contrasting results on the use of TG/HDL ratio (dyslipidemia) as a marker for MetS [35]. A study conducted in California on 258 diabetic participants considered TG/HDL ratio as a strong marker of MetS. However, another study on African Americans showed complementary result on the use of TG/HDL ratio as an ineffective marker of insulin resistance [35, 36]. Our study results are congruent with the previous study on African Americans [36]; we have observed low HDL level in 53% and an elevated triglyceride level in only 40%. This finding can also be the result of ethnic differences.

The present study reveals that MetS was more common among the male participants (66%); this finding is contrary to previous studies which showed a higher prevalence of MetS among women [37]. This change in results can be due to the recent urbanization in our society [22], which has affected males more; as majority of males are now driving cars that have replaced pedal walking, working on desk-based office jobs, sitting at computer for long hours and changing in dietary pattern, that is, junk food, all these have put people at risk of more obesity than it could ever be imagined. 

Despite the close association between MetS components and high blood pressure levels, it is difficult to describe the contribution of each component in the increment of blood pressure levels. Previously, it has been shown that a high prevalence of MetS is present among essential hypertensives than in general population [38]. In Asia Pacific the most contributing component towards development of MetS was hypertension [39], but this is contrary to our findings; in our study 54% of the study participants with uncontrolled blood pressure had MetS. 

The PAMELA study had recently provided us further data on association of MetS with cardiovascular risk factors [40]. Although we have described each component of MetS as an independent risk factor for development of cardiovascular disease, the highest prevalence observed was with a combination of three risk factors, with an extremely low proportion without any risk factor, that is, 2.6%. Increasing research over contribution of MetS components in the development of cardiovascular disease is being done [41]. 

Several epidemiological studies and clinical medicine had recognized that family history is a risk factor for many chronic diseases such as T2DM [42, 43]. Recognition of family history is not only useful in predicting genetic susceptibility of an individual to a disease but is also advantageous for early detection and preventive strategies. Likewise, family history of diabetes is not only a risk factor but is also associated with risk-reducing behaviors [42]. It is well known that T2DM has a strong genetic component and most patients of T2DM have a first-degree relative with diabetes [44]. Many studies have provided evidence for association of MetS with family history of diabetes [42–44]. A nationally representative sample of US adults had shown a significant and positive association between family history of diabetes and MetS components [44]. Another recent cross-sectional study conducted by Das et al. in Kolkata, India, on 448 adults has found that those individuals whose parents and siblings had history of T2DM had more prevalence of MetS components as compared to their counterparts (*P* < 0.001) [45]. This study results are also congruent to other studies as those patients whose parents and/or their siblings had history of diabetes were two (AOR: 2.4, 95% CI: 1.0–4.8) and three times (AOR: 3.0, 95% CI: 1.6–6.0) at risk of developing MetS, respectively.

Epidemiological studies have considered educational status as a surrogate measure for socioeconomic status [46, 47]. However, it can roughly predict the job opportunities and future socioeconomic status. In addition, it plays a vital role in shaping the attitudes and behavior of individual towards health. Studies had found inverse and strong relationship between educational status and presence of MetS [45–47]. There is obviously no direct association of education with MetS but it may influence the life style changes. A study conducted on 300 women also concluded that the risk of MetS increases among less educated individuals [48]. This is also consistent with the current study results as the risk of MetS is 3 times higher in those who have primary-level education, as compared to those with higher level of education. 

The association of education and socioeconomic status with MetS was most observed among females as they are more subjective to socioeconomic inequalities in our continent [46, 49]. Nevertheless, the health of women in Pakistan is compromised, since they are not empowered to take their own decisions and are not allowed to go outside their homes without accompanying male member of the house [49]. This leaves them more exposed to sedentary life style and obesity, precursors of MetS [49]. We do not have the actual income data of the participants so we cannot comment on the direction of association between socioeconomic status and MetS. However we can assume that the population coming to the study setting would be affluent (through cost of examination) and their health behavior and attitude are different from the general population, another reason for high prevalence of MetS.

### 4.1. Role of Family Physician

With the rise of epidemics of type 2 diabetes and CVD worldwide it is important for family physicians to identify those individuals with metabolic syndrome early, so that lifestyle interventions and treatment may prevent the development of diabetes and/or cardiovascular disease. These can be done in three ways; *educational strategies*: through information about MetS, its components, risk factors that are found to be common in Pakistani population; *preventive strategies*: screening for high risk groups such as family history of diabetes, obesity, and so forth, promoting life style changes to avoid obesity; *pharmacotherapy:* Aggressive pharmacologic management of risk factors in preventing complications of T2DM.

### 4.2. Limitations

This study has some limitations. Since this was a retrospective study so temporal associations cannot be ruled out. Moreover, we did not have information on waist circumference of the patients so we could not observe the association of MetS and abdominal obesity (common in Pakistani population) in our study. The data has been extracted from a hospital so extrapolation of the results to the general population must be done with caution. The data was gathered from executive clinics so their health-seeking behavior and patterns can be different from those of the general population. The missing data in the study were handled through mean imputation which can distort the association between the factors. 

## 5. Conclusion

This study from one of the largest cosmopolitan cities of the world highlights a high prevalence of MetS and its components. The presence of such factors among apparently healthy individuals is a cause of concern and requires the need for urgent interventions in order to prevent and control MetS and its associated risk factors such as T2DM and CVD. These interventions should be based on the integrated lifestyle approach, keeping in mind the socioeconomic status of the population. Health care providers should provide awareness regarding MetS risk factors and its prevention to patients and their families. Awareness sessions should also be held to educate the masses through media. Nevertheless, further research is required to explore this vital issue in detail and to develop and test the interventions. 

## Figures and Tables

**Table:** (a) Total population

Parameter	Total population	Men only	Women only	*P* value
	*N* = 1329	*N* = 936	*N* = 393	
Age*	47.6 (±11.6)*	936 (70.5)	393 (29.5)	0.13
% BMI: ≥ 25 kg/m^2^	934 (70.3)	654 (70.0)	280 (30.0)	0.61
% raised triglycerides	532 (41.6)	399 (75.0)	133 (25.0)	0.02
% reduced HDL cholesterol	715 (58.7)	478 (66.9)	237 (33.1)	<0.001
% raised blood pressure	729 (54.9)	536 (73.5)	193 (26.5)	0.006
% raised plasma fasting glucose	834 (63.4)	624 (74.8)	210 (25.2)	<0.001

*Mean (standard deviation), *N*: number (percentage).

*P*-value: difference in males and females, significance at <0.05.

BMI: ≥ 25 kg/m^2^.

Raised triglycerides: ≥150 mg/dL (1.69 mmol/L).

Reduced HDL cholesterol: <40** **mg/dL (1.04 mmol/L) in men, <50 mg/dL (1.29 mmol/L) in women.

Raised blood pressure: ≥130/85 mm Hg.

Raised plasma fasting glucose: ≥100 mg/dL (≥6.1 mmol/L).

**Table:** (b) Age less than 40 years

Parameter	Total population	Men only	Women only	*P*-value
	*N*(%)	*N*(%)	*N*(%)	
Age < 40 years	323 (24.3)	222 (68.7)	101 (31.3)	0.44
BMI ≥ 25 kg/m^2^	218 (67.5)	157 (72.0)	61 (28.0)	0.04
Raised triglycerides	109 (34.8)	131 (75.2)	73 (24.8)	0.03
Reduced HDL cholesterol	195 (63.7)	128 (65.6)	67 (34.4)	0.04
Raised blood pressure	127 (39.3)	105 (82.7)	22(17.3)	<0.001
Raised plasma fasting glucose	135 (42.2)	108 (80.0)	27 (20.0)	<0.001

*N*: number (percentage).

*P*-value: difference in males and females, significance at <0.05.

**Table:** (c) Age more than 40 years

Parameter	Total population	Men only	Women only	*P*-value
	*N*(%)	*N*(%)	*N*(%)	
Age > 40 years	1006 (75.7)	714 (71.0)	292 (29.0)	0.44
% BMI: ≥ 25 kg/m^2^	716 (71.2)	497 (69.4)	219 (30.6)	0.05
% Raised triglycerides	423 (43.8)	317 (74.9)	106 (25.1)	0.02
% Reduced HDL cholesterol	520 (57.0)	350 (67.3)	170 (32.7)	<0.001
% Raised blood pressure	602 (59.8)	431 (71.6)	171 (28.4)	0.59
% Raised plasma fasting glucose	699 (70.2)	516 (73.8)	183 (26.2)	0.002

*N*: number (percentage).

*P*-value: difference in males and females, significance at <0.05.

**Table 2:** Univariate and multivariable logistic regression analysis of factors associated with MetS.

Variable	MS present *N* = 847 (63.7)	MS absent *N* = 482 (36.3)	Unadjusted odds ratio (95% C.I)	Adjusted odds ratio (95% CI)	*P* value
Gender					
Female	227 (57.8)	166 (42.2)	1	1	0.003
Male	620 (66.2)	316 (33.8)	1.4 (1.1–1.8)	2.1 (1.6–3.2)	
Educational status^€^					
University level	242 (28.6)	97 (20.2)	1	1	
Secondary level	255 (30.0)	179 (37)	1.5 (1.3–1.8)	2.2 (1.5–3.2)	0.01
Primary level	350 (41.4)	206 (42.8)	2.0 (1.4–2.8)	3.0 (2.4–3.8)	
Past history of diabetes					
No	483 (57)	294 (61)	1	1	<0.001
Yes	364 (43)	188 (39)	3.3 (2.20–4.6)	5.3 (1.0–74)	
Family history of diabetes of parents					
No	338 (40.0)	146 (30.4)	1	1	0.04
Yes	509 (60.0)	336 (69.6)	2.3 (1.2–4.0)	3.0 (1.6–6.0)	
Family history ofdiabetes of sibling					
No	295 (34.8)	145 (30)	1	1	0.03
Yes	552 (65.1)	337 (70)	1.4 (1.2–1.6)	2.4 (1.0–4.8)	
Family history of CVD of parents					
No	232 (27.5)	366 (76.1)	1	1	NS
Yes	615 (72.5)	116 (23.9)	1.5 (0.5–4.5)	6.9 (0.8–8.4)	
Family history of CVD of sibling					
No	289 (34.2)	184 (38.2)	1	1	NS
Yes	558 (65.8)	298 (61.8)	4.4 (0.8–6.4)	6.0 (4.7–7.8)	

95% CI: 95% confidence interval, NS: not significant at *P* value <0.05. Educational Status^€^: university level (14 years or more), secondary level (6–12 years of education), and primary level (1–5 years of education).
